# Supplementary material for: Digestibility of insect meals for Pacific white shrimp (Litopenaeus vannamei) and their performance for growth, feed utilization and immune responses
Source: PLoS One. 2021 Nov 19;16(11):e0260305. doi: 10.1371/journal.pone.0260305 (PMC8604367; doi:10.1371/journal.pone.0260305)
Supplement: Attachment — Submitted filename: Response to Reviewers.docx [file pone.0260305.s001.docx]

**Dear Editor In-Chief,**

Thank you very much for the reviewer’s comments to improve our manuscript (PONE-D-21-14702). According to the reviewer’s comments/suggestions including yours, all the required changes or revisions were carefully made in the revised manuscript. The main corrections and answers are listed below:

**[Journal requirements]**

**Q1:** Please ensure that your manuscript meets PLOS ONE's style requirements, including those for file naming. The PLOS ONE style templates can be found at https://journals.plos.org/plosone/s/file?id=wjVg/PLOSOne_formatting_sample_main_body.pdf and https://journals.plos.org/plosone/s/file?id=ba62/PLOSOne_formatting_sample_title_authors_affiliations.pdf.

**A1:** We corrected our manuscript very carefully based on the Journal’s requirements. Please check the revised manuscript.

**Q2:** We note that the grant information you provided in the ‘Funding Information’ and ‘Financial Disclosure’ sections do not match.
When you resubmit, please ensure that you provide the correct grant numbers for the awards you received for your study in the ‘Funding Information’ section.

**A2:** We corrected it. Please find the ‘Funding Information’ section in our submission.

**Q3:** We note that you have indicated that data from this study are available upon request. PLOS only allows data to be available upon request if there are legal or ethical restrictions on sharing data publicly. For more information on unacceptable data access restrictions, please see http://journals.plos.org/plosone/s/data-availability#loc-unacceptable-data-access-restrictions. In your revised cover letter, please address the following prompts:
a) If there are ethical or legal restrictions on sharing a de-identified data set, please explain them in detail (e.g., data contain potentially sensitive information, data are owned by a third-party organization, etc.) and who has imposed them (e.g., an ethics committee). Please also provide contact information for a data access committee, ethics committee, or other institutional body to which data requests may be sent.
b) If there are no restrictions, please upload the minimal anonymized data set necessary to replicate your study findings as either Supporting Information files or to a stable, public repository and provide us with the relevant URLs, DOIs, or accession numbers. For a list of acceptable repositories, please see http://journals.plos.org/plosone/s/data-availability#loc-recommended-repositories.
We will update your Data Availability statement on your behalf to reflect the information you provide."

**A3:** There are no restrictions on sharing data publicly in our data. We attached standard deviation in Table 6, Table 7 and Table 10.

**Q4:** Your ethics statement should only appear in the Methods section of your manuscript. If your ethics statement is written in any section besides the Methods, please delete it from any other section.

**A4:** We separated it and presented as an ‘Ethics statement’ section in “Material and methods” **(Line 81–85)**.

**[Editor In-Chief]**

**Q1:** All tables should stand alone with full definition of all abbreviations.

**A1:** We revised all the Tables as the editor suggested. Please find the revised Tables in the revised manuscript.

**Q2:** Make sure that all works cited in the text are in the reference list, that the presentation is consistent and that correct information is given.

**A2:** We carefully checked all the references and corrected accordingly.

**Q3:** Define and explain all acronyms and abbreviations on first mention in the text.

**A3:** We carefully revised the manuscript and confirmed that all the acronyms and abbreviations are fully explained in its first mention.

**Q4:** On first mention of a species in the text give both the common (trivial) and formal name, and make sure that the presentation is correct and consistent.

**A4:** We carefully revised the manuscript and corrected the mistakes.

**Q5:** Make sure that symbols, sub- and super-scripts, upper- and lower-case are presented correctly, and that there is correct and consistent use of italics, brackets and punctuation etc.

**A5:** We corrected and made them constant. Please check the revised manuscript.

**Q6:** There are mistakes in the reference list, including incorrect reporting, inconsistent presentation, spelling mistakes and problems with use of punctuation etc.

**A6:** We carefully revised and corrected the reference list including mistakes along with journal’s requirements.

**[Reviewer #1]**

**Q1:** Line 19. Wrong conjugation, change "was" to "were".

**A1:** We corrected the sentence as the reviewer suggested **(Line 19)**.

**Q2:** Line 24-25. It is not clearly understood how insect meals were included, please rephrase to reflect that 17% of tuna by-product meal was supplemented with 10% insect meal.

**A2:** We agree to the reviewer’s comment. We modified the sentence as follows; ‘For a feeding trial, a control diet was formulated using 27% tuna byproduct meal as a fish meal source and seven other diets were prepared replacing 10% tuna byproduct meal in the control diet with each insect meal (10%).’ **(Line 24–26)**.

**Q3.** Line 38. "Insect also provides" or "insects also provide".

**A3:** We corrected it as the reviewer suggested **(Line 36)**.

**Q4.** Line 42. "day matter", please change this.

**A4:** We revised the typo-error in the sentence as the reviewer suggested **(Line 40)**.

**Q5.** Line 47. How do the insect EAA profiles significantly differ from fish meal? What do previous findings show on this matter?

**A5:** According to many previous studies, fish meal was the best ingredient for fishes and shrimps because of its excellent essential amino acid balance. The essential amino acid balance in the insects has also been reported to be comparable to those of fish meal with minor difference (Nogales‐Mérida et al., 2019). Therefore, we changed the sentence to ‘Insects are rich in essential amino acids (AAs) making them highly desirable as an excellent protein sources for aquaculture [1]’. **(Line 43–44)**.

**Q6.** Line 144. "Seven other diets"

**A6:** We corrected the sentence as the reviewer suggested **(Line 150)**.

**Q7.** Line 148. Is there a particular reason for the four feedings and also the range in feeding rate (3-10%)? Are they fed more as the trial progresses or are they fed more based on observed feed intake?

**A7:** The feeding times of four have been the most frequently used feeding time for shrimp (Tacon et al., 2002). The average feeding rate for shrimp trials has long been approximately 2–10% of total body mass (Tacon et al., 2002). We fed the shrimp in the trial with the amounts as follows; 8–10% (0.17–2 g), 5–7% (3–6 g) and 3–4% (>7 g) based on the growth rate of shrimp. The feeding rate was reduced as the trial progressed. We added the details in the revised manuscript **(Line 155–156)**.

References: Tacon AGJ, Cody JJ, Conquest LD, Divakaran S, Forster IP, Decamp OE. Effect of culture system on the nutrition and growth performance of Pacific white shrimp (*Litopenaeus vannamei* Boone) fed different diets. Aquaculture nutrition 2002; 8:121–137.

**Q8.** Line 150. Please remove the "in" before every three days.

**A8:** We changed the sentence as the reviewer suggested **(Line 157)**.

**Q9.** Line 151. Please change "measure" to "measured".

**A9:** We changed the sentence as the reviewer suggested **(Line 158)**.

**Q10.** Line 316. "been" should be removed.

**A10:** We changed the sentence as the reviewer suggested **(Line 321)**.

**Q11.** Line 330-331. What makes you say that excess dietary chitin can impair growth and feed utilization? Has there been a study to back this or is it speculation based on experience that excess of most things tend to be detrimental to the cultured species' health?

**A11:** It was reported that dietary chitin supplementation at proper levels (2–5% in diet) enhances growth, feed utilization and digestibility of shrimp (Shiau and Yu, 1998). However, an excessive dietary chitin supplementation (10% in diet) had a negative effect on their growth, feed utilization and digestibility (protein and lipid) in the study. We modified the sentence as follows: ‘Nonetheless, dietary chitin levels should be carefully optimized, as excessive dietary chitin supplementation (>10% in diet) reduced growth, feed utilization and digestibility (protein and lipid) of *P. monodon* [39].’ **(Line 335–337)**.

References: [39] Shiau SY, Yu YP. Chitin but not chitosan supplementation enhances growth of grass shrimp, *Penaeus monodon*. J Nutr. 1998; 128:908–912.

**Q12.** Line 332. I am unclear what is meant by this statement, it seems to mean that 37% of FM was completely replaced and not supplemented with insect meal, please re-word or please explain what is meant here.

**A12:** We agree with the reviewer’s comment. The sentence could make the readers confused even though fish nutritionists usually use the actual replacement percentage from FM, which is 37% fish meal replacement in this study for 10% fish meal in the diet (10/27 =0.37) with 10% insect meal. Nonetheless, we modified the sentence to improve readability or understanding as the reviewer suggested as follow; ‘In the feeding trial, dietary replacement of 17% FM with each insect meal did not show any significantly reduced growth or feed utilization of *L. vannamei*.’ **(Line 338–339)**.

**Q13.** Line 359. I do believe that there is more than one finding, so the correct term should be "findings", even if you are only referring to that particular section.

**A13:** Thank you for the suggestion. We corrected the sentence accordingly **(Line 365)**.

**[Reviewer #2]**

**Q1**. Manuscript must be check for grammar structure by native person.

**A1.** The manuscript was rechecked by an English native (essayreview.co.kr). We carefully revised the original manuscript to improve readability. Please find the revised manuscript.

**Q2.** Discussion part should be modified and use newer references.

**A2.** We agree with the reviewer’s comment. We carefully revised some sentences accordingly in discussion part. Please check the revised manuscript.

**Q3.** Material and methods must explain in more details.

**A3.** We agree to the reviewer’s comment. We added more details in material and methods section including detailed feeding rate. Please check the revised manuscript.
