# Supplementary material for: Digestibility of insect meals for Pacific white shrimp (Litopenaeus vannamei) and their performance for growth, feed utilization and immune responses
Source: PLoS One. 2021 Nov 19;16(11):e0260305. doi: 10.1371/journal.pone.0260305 (PMC8604367; doi:10.1371/journal.pone.0260305)
Supplement: Attachment — Submitted filename: Response to Reviewers.docx [file pone.0260305.s002.docx]

**Dear Editor In-Chief,**

Thank you very much for the reviewer’s comments to improve our manuscript (PONE-D-21-14702R1). According to the reviewer’s comments/suggestions including yours, all the required changes or revisions were carefully made in the 2^nd^ revised manuscript. The corrections and answers are listed below:

**[Journal requirements]**

**Q1:** Please review your reference list to ensure that it is complete and correct. If you have cited papers that have been retracted, please include the rationale for doing so in the manuscript text, or remove these references and replace them with relevant current references. Any changes to the reference list should be mentioned in the rebuttal letter that accompanies your revised manuscript. If you need to cite a retracted article, indicate the article’s retracted status in the References list and also include a citation and full reference for the retraction notice.

**A1:** We checked each reference very carefully once again and found that there was no retracted paper in the reference list.

**[Reviewer #1]**

**Q1:** Line 26. There is no need to repeat “(10%)”

**A1:** We corrected the sentence as the reviewer suggested **(Line 26)**.

**Q2.** Line 114–115. Note to authors and/editors to ensure that “–25” is on the same line

**A2:** We dried the experimental diets after pelleting at “25℃” and stored the diets at” –25℃”.

**Q3.** Line 178–. “were weight” is the correct grammatical form.

**A3:** We changed the sentence as the reviewer suggested **(Line 178)**.

**Q4.** Line 332–. "have"

**A4:** We changed the sentence as the reviewer suggested **(Line 331)**.

**Q5.** Line 354–. "which have"

**A5:** We changed the sentence **(Line 351)**.

**Q6.** Line 361–. "which are"

**A6:** We changed the sentence as the reviewer suggested **(Line 358)**.

**Q7.** Line 389–. "decreases"

**A7:** We changed the sentence as the reviewer suggested **(Line 386)**.

**Q8.** Line 401–. Rather than "activities," would “properties” be a better word?

**A8:** We changed the sentence as the reviewer suggested **(Line 399)**.

**Q9.** Line 412/413– “and to assess”

**A9:** We changed the sentence as the reviewer suggested **(Line 409)**.

**[Reviewer #2]**

**Q1**. Manuscript should be prepared as a short paper and must check by native people.

**A1.** The manuscript was rechecked by an English native (essayreview.co.kr) one more time. We carefully revised the manuscript (PONE-D-21-14702R1) to improve readability. Please find the revised manuscript (R2).

**Q2.** Authors should update references in the introduction and discussion parts and explain the reasons of observed results.

**A2.** We agree with the reviewer’s comment. We updated references and carefully revised some sentences accordingly in introduction and discussion sections. Please check the revised manuscript.
